# Could the Gut Microbiota Serve as a Therapeutic Target in Ischemic Stroke?

**DOI:** 10.1155/2021/1391384

**Published:** 2021-04-17

**Authors:** Jiyao Zhang, Qiang Tang, Luwen Zhu

**Affiliations:** ^1^Graduate School, Heilongjiang University of Chinese Medicine, 24 Heping Road, Xiangfang District, Harbin 150040, Heilongjiang, China; ^2^Rehabilitation Center, Second Affiliated Hospital of Heilongjiang University of Chinese Medicine, 411 Guogeli Street, Nangang District, Harbin 150001, Heilongjiang, China; ^3^Brain Function and Neurorehabilitation Laboratory, Second Affiliated Hospital of Heilongjiang University of Chinese Medicine, 411 Guogeli Street, Nangang District, Harbin 150001, Heilongjiang, China

## Abstract

The brain-gut axis is a relatively recent discovery of a two-way regulation system between the gut and brain, suggesting that the gut microbiota may be a promising targeted prevention and treatment strategy for patients with a high risk of acute cerebral ischemia/reperfusion injury. There are many risk factors for ischemic stroke, and many studies have shown that the gut microbiota affects the absorption and metabolism of the body, as well as the risk factors of stroke, such as blood pressure, blood glucose, blood lipids, and atherosclerosis, either directly or indirectly. Furthermore, the gut microbiota can affect the occurrence and prognosis of ischemic stroke by regulating risk factors or immune responses. Therefore, this study aimed to collect evidence of the interaction between gut microbiota and ischemic stroke, summarize the interaction mechanism between the two, and explore the gut microbiota as a new targeted prevention and treatment strategy for patients with high ischemic risk.

## 1. Introduction

Ischemic stroke (IS) refers to a group of diseases that cause ischemia and hypoxia, irreversible damage, and brain tissue necrosis [1]. IS is characterized by a high morbidity, lethality, disability, and cure rate. At present, the clinical treatment strategies of ischemic stroke mainly involve improving blood supply and protecting the structure and function of nerve cells, of which the means of improving blood supply include thrombolysis, dilation of blood vessels, vascular remodeling, establishment of collateral circulation, and regulation of blood state, while the means of protecting the structure and function of nerve cells are mainly drugs [2–4]. But the treatment is limited by a short timeframe, and the associated complications are complex; thus, early prevention, treatment, and improvement of prognosis are of great significance for improving the quality of life in patients with IS. According to statistics, 50% of stroke patients experience gastrointestinal complications in the clinic, including dysphagia, gastrointestinal bleeding, constipation, and intestinal incontinence [5]. Patients with cerebrovascular disease associated with gastrointestinal complications often have a poor prognosis and may experience worsening neurological function and even increased mortality [6].

The gut microbiota refers to a collective community of microorganisms in the gastrointestinal tract including more than 100 trillion microorganisms, of which the number of bacteria is up to 10^14^; it is a considerable metabolic organ in the human body and is of great significance for maintaining homeostasis [7, 8]. The intestinal microecological system, comprising the gut microbiota, plays an indispensable role in the energy and nutrition supplementation of the body. It also helps to fight infection by driving the immune system to mature and maintaining the stability of the immune response [9] through mechanisms such as degradation of polysaccharides, decomposition of indigestible foods and starch in the daily diet [10], improvement of host immune function [11], enhancement of intestinal motility, and regulation of brain development [12, 13].

The gut microbiota includes anaerobic bacteria, aerobic bacteria, and facultative anaerobic bacteria, of which *Firmicutes* and *Bacteroides* are in the highest abundance, accounting for 70–75% of the healthy bacteria, and *Proteobacteria*, *Actinomycetes*, *Fusobacterium*, and *Verrucomicrobia* account for a small proportion [14]. The gut microbiota can be divided into beneficial, harmful, and two-way bacteria, according to their relationship with the host; homeostasis is closely related to the balance of bacteria within the human internal environment. Beneficial bacteria are the main body of the gut microbiome and commonly include *Bifidobacterium* and *Lactobacillus*, which aid in metabolism and barrier protection in the host. Common harmful bacteria include *Salmonella* and pathogenic *Escherichia coli*, and the ratio of beneficial bacteria to harmful bacteria is related to the occurrence of many diseases of the human body.

Construction of the human gut microbiome is affected by environmental and genetic factors, where the diet accounts for 57% of its composition [15]. In microbiology, the ratio of *Firmicutes* to *Bacteroides* is usually used as a sign of gut disturbance, which includes the diversity, uniformity, and abundance of the gut microbiota. Various factors affect the homeostasis of the gut microbiota, including infection, stress, trauma, hypoxia, food, drugs, and physical and chemical stimulation [16]. Gut microbiota imbalance is considered an important cause of various diseases, such as cardiovascular diseases and hypertension [17–19], and is closely related to the occurrence and development of central nervous system diseases [20–24].

The gut microbiota also plays an important role in the two-way communication between the central and intestinal nervous systems, i.e., the brain-gut-microbiota axis. For example, it was found that gut microbiota disorders caused changes in brain-derived neurotrophic factors in the hippocampus of mice [25]. Short-chain fatty acids (SCFAs) were used as messengers to help microglia with repair function to rapidly respond to inflammatory responses after reaching the brain through blood circulation [26]; simultaneously, the gut microbiota stimulated the intestinal endocrine cells to secrete neuroactive molecules, such as *γ*-aminobutyric acid and 5-hydroxytryptamine (5-HT), which affect the central nervous system after entering the blood [27]. Liu et al. administered *Clostridium butyricum* via gavage to model mice with vascular dementia, reporting that their cognitive function and cerebrovascular pathological changes were improved [28]. Additionally, studies have shown that gut microbiota disorders are closely related to Alzheimer's disease [29], Parkinson's disease [30, 31], stroke [32–37], and autonomic spectrum disorder [38, 39].

Pathways involved in the two-way regulation system between the intestine and brain may include the intestinal immune system, gut microbiota metabolism system, intestinal mucosal barrier, blood-brain barrier, neuroanatomical pathways between the brain and intestine, and hypothalamic-pituitary-adrenal axis pathways [40]. The gut microbiota interacts with the brain through the immune system, vagus nerve, and neurotransmitters [11, 32]; the exact mechanism has not been fully elucidated. The mainstream view is that the nervous, immune, and endocrine systems all use the vagus nerve as their main pathway, thereby participating in brain-gut-microbiota axis regulation [41–43].

Animal experiments have shown that probiotics, such as *Bifidobacterium longum*, improve anxiety and depression in mice through the vagus nerve [44, 45]. Secretory cells on the surface of the intestinal mucosa regulate the levels of body-related hormones, such as gastrin and adrenocortical hormones, and affect brain function [46]. The gut microbiota regulates a series of neurotrophic factors or proteins related to brain development and plasticity, such as brain-derived neurotrophic factors, synaptophysin, and postsynaptic dense area proteins [47]. Compared with common animals, the blood-brain barrier permeability of germ-free animals was increased, and the microglia differed in both morphology and function in animal experiments [37].

An increasing amount of data indicates that the gut microbiota is a crucial factor in the development, sequelae, and treatment of stroke. IS changes the composition of the gut microbiota; these changes typically affect the immune balance of hosts, increase ischemic brain damage, or exert neuroprotective effects. Although most studies regarding the association between the gut microbiota and stroke are derived from animal models and patients, various therapeutic targets have been identified.

**Figure 1:**
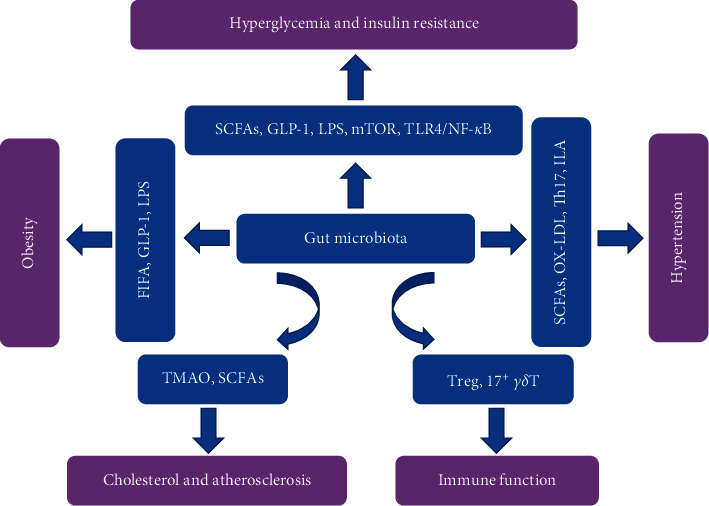
The relationship between the gut microbiota and risk factors related to IS. FIAF: fasting-induced adipose factor; GLP-1: glucagon-like peptide-1; ILA: indole lactic acid; IS: ischemic stroke; LPS: lipopolysaccharide; mTOR: mammalian target of rapamycin; NF-*κ*B: nuclear factor kappa B; OX-LDL: oxidized low-density lipoprotein; SCFAs: short-chain fatty acids; Th17: T helper cell 17; TLR4: Toll-like receptor 4; TMAO: trimethylamine oxide; Treg: regulatory T cells.

The gut microbiota can modulate the risk factors for stroke and play a role in its development [48–50]. As shown in Figure 1, a comprehensive understanding of the mechanism of interaction between the gut microbiota and IS will provide new ideas and directions for the prevention and treatment of IS.

## 2. Risk Factor Studies Linking the Gut Microbiota to IS

### 2.1. Obesity

The World Health Organization predicts that the global number of overweight individuals will reach 2.16 billion by 2030 [31]. Obesity refers to a disorder of the body's energy metabolism that affects the occurrence of many diseases, such as type 2 diabetes, stroke, and cardiovascular disease. The gut microbiota is the largest microecosystem of the human body, affecting physical energy and metabolism; found in the human intestinal tract, it decomposes polysaccharides, oligosaccharides, and glycoproteins that are not easily digested. For example, the gut microbiota digests dietary fiber and converts it into monosaccharides and SCFAs, providing nutrition, energy, and signal transduction to the host. Evidence has shown that the gut microbiota helps the host to fully absorb the energy in food by affecting the lipid metabolism process of the body, either directly or indirectly; additionally, it plays an important role in the pathophysiology of obesity [51, 52].

Changes in the gut microbiota cause disorders in lipid metabolism and increases blood viscosity, further resulting in cerebral arteriosclerosis and even IS. It was also found that gut microbiota inhibited fasting-induced adipose factor and increased fat storage [53]. Additionally, methane produced by methanogens in the intestinal tract slows down peristalsis [54, 55] and increases the absorption of nutrients in the intestine, leading to obesity. Kalliomäki et al. [56] found that small differences in fecal microbial composition during childhood could better predict adult body mass. Bäckhed et al. confirmed, for the first time, that the gut microbiota was closely related to insulin resistance in obesity through animal experiments [52, 53, 57]. During these experiments, the gut microbiota of ordinary mice was transplanted into germ-free mice. As a result, the mice exhibited an increased fat content and developed insulin resistance; it was later discovered that germ-free mice did not appear obese after a high-fat, high-sugar diet. Moreover, Turnbaugh et al. [52] showed that if the gut microbiota of obese and lean mice were implanted into the intestines of germ-free mice, the amount of fat produced by the gut microbiota of obese mice was significantly higher than that produced by the lean mice. Additionally, both casein peptide and glucagon-like peptide-1 (GLP-1), secreted by intestinal L cells, can suppress appetite [58, 59].

Chang et al. found that the level of *Proteobacteria* and the ratio of *Firmicutes* and *Bacteroides* were increased in mice fed a high-fat diet [60]. Caused by gut microbiota imbalance, intestinal barrier dysfunction activates the inflammatory response of adipose tissue in the intestine and peripheral sites [61]. Le et al. confirmed that, compared with the normal population, the abundance of gut microbiota in obese people was significantly reduced and accompanied by an inflammatory phenotype [62]. When disturbances in the gut microbiota occur, the number of beneficial bacteria that protect the intestinal barrier is reduced, whereas the number of harmful bacteria that produce large amounts of endotoxins (lipopolysaccharide (LPS)) increases. LPS enters the bloodstream through the intestinal tract, stimulates the overexpression of Toll-like receptors (TLRs), and activates the nuclear factor kappa B (NF-*κ*B) pathway, peripheral immune response, and chronic inflammatory response [63].

### 2.2. Hyperglycemia and Insulin Resistance

Diabetes is an independent risk factor for IS onset. Compared with ordinary people, the risk of stroke in patients with diabetes is 1.8 to 6 times higher; additionally, the incidence and mortality of adverse events after stroke are significantly increased [64, 65]. Diabetic patients exhibit varying degrees of atherosclerosis, dysfunction of microvascular endothelial cells, thickening of the basement membrane of capillary walls, and blood in a high-viscosity, high-coagulation state, hindering microcirculation. Studies have shown that the gut microbiota is related to the occurrence and progression of diabetes. Nonobese diabetic mice with innate immune deficiency developed diabetes when they were fed in a sterile environment but not during normal feeding; this occurred due to the lack of normal intestinal bacteria in the sterile environment [66].

Qin et al. found that gut microbiota disorders are common in patients with type 2 diabetes [67]. Metagenomic analysis showed that the number of Gram-positive bacteria in the gut microbiota of patients with type 2 diabetes was significantly reduced, and the proportion of Gram-negative bacteria increased [68]. Results indicated that the blood glucose level in the human body was negatively correlated with the number and proportion of beneficial bacteria and positively correlated with *Enterococcus* [69]. Bacteria that inhibit the effects of diabetes include *Eubacteria rectale*, *Roseburia*, *Verrucomicrobia*, *Clostridium*, *Faecalibacterium prausnitzii*, and *Akkermansia muciniphila*; bacteria that promote the effects of diabetes include *E*. *coli*, *Bacteroides stercoris*, *Desulfovibrio*, *Clostridium mutans*, *Streptococcus mutans*, *Lactobacillus gasseri*, and *Haemophilus parainfluenza* [67, 70].

Gut microbiota imbalances induce lower levels of SCFAs, the reason being that SCFAs affect the secretion of GLP-1 and casein peptides by intestinal mucosa L cells. GLP-1 is an intestinal insulinotropic, whereas casein peptides play an important role in the regulation of blood glucose homeostasis [71, 72]. Many studies have shown that intestinal immune homeostasis due to gut microbiota disturbances and intestinal mucosal dysfunction due to inflammatory activation are closely related to the development of insulin resistance [73, 74]; gut microbiota imbalances trigger inflammatory responses and affect insulin-related signaling pathways, such as the mammalian target of rapamycin signaling pathway and TLR4/NF-*κ*B signaling, thereby causing insulin resistance and elevated blood glucose [27, 75]. Similarly, Navarrete et al. found that LPS stimulated the body to produce a large number of cytokines, triggering the inflammatory response and insulin resistance in the body [76]. On the other hand, Yadav et al. found that probiotic *VSL #3* inhibited butyric acid and GLP-1 in mice, thus, preventing obesity and diabetes [77]. De et al. also demonstrated that SCFAs, propionate and butyrate, activate intestinal glyconeogenesis through complementary mechanisms [78].

### 2.3. Hypertension

The gut microbiota also plays a role in the development of hypertension during brain-gut axis mediation. As the number one risk factor for cardiovascular and cerebrovascular diseases, hypertension causes approximately 9.4 million deaths worldwide per annum. Hypertension is the result of a combination of genetic and environmental factors; its severity and duration are closely related to the onset and prognosis of IS and have a significant triggering effect on stroke [79, 80]. A large number of studies have recently shown that while the gut microbiota plays an important role in the pathogenesis of hypertension, the state of hypertension in the body also affects the intestinal microecological environment and function. Related animal studies confirmed that the gut microbiota of hypertensive rats appeared to be imbalanced; this imbalance manifested following an increase in the ratio of *Firmicutes* and *Bacteroides*, reducing the number of intestinal bacteria that produce acetate and butyrate, and significantly decreasing the diversity, evenness, and abundance of microorganisms [81]. This ecological dysregulation pattern was also confirmed in the microbial community of patients with hypertension.

Mice fecal transplantation experiments have shown that a disordered gut microbiota causes elevated blood pressure [82] and can accelerate vascular immune cell infiltration and inflammatory responses, causing vascular dysfunction and hypertension [83]. Intestinal ecological disorders can increase oxidized low-density lipoprotein (OX-LDL), causing blood vessel constriction and, thus, leading to hypertension [84, 85]. The evidence indicates that a high-salt diet induces T cells to differentiate into CD4^+^ Th17 cells, causing autoimmunity and hypertension. In salt-sensitive hypertensive animal experiments, high-salt diets increased blood pressure and stimulated specific gut microbiota to produce more pathogenic T helper cell (Th) 17 cells. Injecting mice with *Lactobacillus murinus* or *Lactobacillus acidophilus* normalized Th17 cell numbers, reduced blood pressure, and reduced disease severity [86]. Wilck et al. found that a high-salt diet inhibited the metabolism of *L*. *murinus* in the intestine to produce indole lactic acid; this caused T cells to differentiate into Th17, and induced salt sensitivity, which was verified in humans [86].

Related studies have shown that bacterial metabolite SCFAs are involved in the regulation of blood pressure, while the gut microbiota participates in the regulation of blood pressure through the gut-sympathetic nervous system axis [87]. Among the biologically active metabolites are SCFAs, secreted by intestinal microorganisms; acetic acid is related to the development of symptomatic hypotension and vasodilation, while butyric and propionic acid induce colonic arterial dilatation and have vasodilating effects on the tail artery [88–90].

Holmes et al. analyzed populations in Asia and Europe, reporting that SCFAs and blood pressure levels were significantly correlated [91]. Olfactory receptor 78 (Olfr78) and G-protein-coupled receptor 41 (Gpr41) are two SCFA receptors of the human body [92] that regulate blood pressure; both respond to acetate and propionate. Olfr78 acts on the arteriolar arterioles to promote the release of renin [93], produces vasoconstrictive effects, and increases hypertension; Gpr41, mainly expressed in the smooth muscle cells of the kidney and large blood vessels [92], reduces blood pressure. After the SCFA signal (produced by the digestion of dietary fiber by the gut microbiota) enters the blood circulation, Gpr41 is activated to reduce blood pressure; as blood pressure decreases and the level of SCFAs in the blood increases, Olfr78 is triggered to prevent blood pressure from falling.

Santisteban et al. reported that intestinal ecological disorders were both a result and direct cause of increased blood pressure [94]. In their experiments, hypertensive rat models showed increased intestinal wall permeability, increased fibrous and inflammatory markers, and decreased tight junction protein and blood flow. Simultaneously, gut microbiota disturbances enhanced sympathetic nerve transmission between the intestine and the paraventricular nucleus of the hypothalamus. Additionally, the intestinal microbial metabolite trimethylamine oxide (TMAO) was found to promote the development of atherosclerosis and exhibited a certain degree of pressor effect, increasing the occurrence of cardiovascular events [95].

Regulation of blood pressure depends on the degree of vasoconstriction and relaxation, with higher levels of OX-LDL causing hypertension by inhibiting the production of nitric oxide and endothelin-1 [84]. Studies have shown that the intestinal ecological disorder increases OX-LDL and promotes vasoconstriction [85]. In addition, blood pressure reduction can be achieved through the intervention of dysfunctional gut microbiota. Marques et al. found that a high-fiber diet and acetate supplementation reduced blood pressure and improved both myocardial fibrosis and left ventricular hypertrophy in mice [96]. Beneficial bacteria and their metabolites in the intestine improve insulin sensitivity and reduce insulin resistance, vascular inflammation, and blood lipid levels, thereby improving blood pressure.

A meta-analysis including nine clinical trials showed that the consumption of probiotics reduced systolic and diastolic blood pressure in patients, compared with the control group. Additionally, consumption of multiple probiotics was shown to reduce blood pressure more significantly than a single species [97]; this was confirmed by Seppo et al., who reported that the long-term intake of fermented milk containing *Lactobacillus LBK-16H* could lower blood pressure [98]. By increasing the production of 5-HT, dopamine, and norepinephrine, as well as the excretion of urine sodium, the gut microbiota were able to reduce the incidence of salt-sensitive hypertension [99].

### 2.4. Cholesterol and Atherosclerosis

In addition to hypertension, obesity, and hyperglycemia, important risk factors affecting IS include changes in craniocervical aortic atherosclerosis (AS), while hyperlipidemia is an important influencing factor. Evidence has shown that a long-term, high-fat diet might alter the composition of the gut microbiota, whereas hyperlipidemia could affect its reproduction and metabolism; additionally, the gut microbiota, a high-fat diet, and hyperlipidemia were reported to be closely linked [100–103].

It is currently believed that the gut microbiome plays an important role in atherosclerotic diseases such as intestinal bacterial, and other infections that aggravate plaque development or trigger plaque rupture and aggravate the effect of intestinal microflora metabolites on plaque [104–106]. Grill et al. found that the cholesterol level in fecal excretion from normally colonized animals was higher than that of germ-free animals; similarly, the cholesterol level in the blood of normally colonized animals was twice that of germ-free animals after receiving a high cholesterol diet [100]. Evidence suggests that the presence of intestinal bacterial DNA is found in plaques [104], and it has additionally been reported that the intestinal metagenome of patients with symptomatic AS is rich in genes encoding peptidoglycan synthesis, which promotes the formation of AS by activating immune system functions [107].

TMAO is an important metabolite of the gut microbiota that may affect AS formation [108]; it is produced when the gut microbiota decomposes choline-rich foods into trimethylamine, which is oxidized through flavin-containing monooxygenases in the liver [19, 109]. Studies have shown that the mechanisms by which TMAO promotes AS may include inhibiting reverse cholesterol transport [95], promoting the expression of macrophage scavenger receptors CD36 and SRA, increasing foam cell formation [110], and promoting platelet hyperresponsiveness and thrombosis [111]. Wang et al. found that the AS plaque area in the aortic root of ApoE-/-mice positively correlated with the concentration of TMAO in plasma [110]. When trimethylamine lyase activity was inhibited by 3,3-dimethyl-1-butanol, TMAO levels in the plasma of ApoE-/-mice, as well as AS plaque area, decreased significantly [112]. In animal experiments, the direct supply of TMAO precursors (choline, carnitine, and *γ*-butyrobetaine) also promoted the development of AS [113].

Bile acids are primarily used to eliminate excess cholesterol in the body and help improve AS. Studies have shown that anaerobes and facultative anaerobes in the large intestine synthesize primary bile acids into secondary bile acids; after entering the blood, these regulate the metabolism of lipids and glucose in the body through nuclear and G-protein-coupled receptors [114–116]. In addition, butyrate, an SCFA, significantly reduced the expression of genes such as NF-*κ*B, INF-*γ*, TLR, and tumor necrosis factor alpha (TNF-*α*), inhibited the inflammatory response [117], and slowed the development of AS. It is also believed that *L*. *acidophilus* and *Bifidobacterium* reduce total cholesterol and low-density lipoprotein levels in patients with hypercholesterolemia [118]. Other metabolites of the gut microbiota, such as indole derivatives and polyamines, also have an inhibitory effect on AS [119, 120].

### 2.5. Immune Function

The inflammatory response, mainly mediated by the peripheral immune T lymphocytes, is an important component in the pathophysiological process of IS [121]. After the occurrence of stroke, the immune system is activated to participate in brain injury caused by ischemia; however, it is also limited by the immunosuppressive effect of the brain, to prevent further aggravation of the injury. The largest gathering place for immune cells is the human gastrointestinal tract; still, the immune system and gut microbiota are essential for the growth and development of immune cells and maintenance of normal functions [122].

A gut microbiota imbalance affects regulatory T (Treg) and interleukin (IL)-17^+^*γδ*T cells and participates in cerebral ischemic injury [121]; *γδ*T cells are primarily located on the epithelial surface of the intestinal tract and mainly perform innate immune functions [123]. Studies have shown that when the gut microbiota are imbalanced, *γδ*T cells secrete a large amount of IL-17 and have a chemotactic effect on neutrophils and monocytes, thereby aggravating ischemic brain injury [124]. By contrast, Treg cells secrete IL-10, inhibit postischemic inflammation [125, 126], and have been reported to exert neuroprotective effects by regulating the peripheral immune system, rather than acting directly on damaged brain tissue [127].

### 2.6. Animal Model Studies Linking Gut Microbiota to IS

Stroke directly affects the composition of the gut microbiota, by either indirectly changing the intestinal microenvironment or generating a large number of molecular signals [32, 128]. It was found that, following stroke, the diversity of the fecal flora decreased, its composition and structure changed significantly, and the relative abundance of *Prevotella* decreased; all changes related to neuronal injury and apoptosis in the ischemic area [129]. Increased intestinal permeability, thus, allows the gut microbiota, metabolites, cytokines, and chemokines to enter the blood and cross the blood-brain barrier into the brain, thereby affecting brain function [130].

When the gut microbiota is imbalanced, the increased blood volume of LPS induces the microglia to polarize to the M1 type and promotes the release of proinflammatory cytotoxins. This inhibits the activation of microglia and improves the pathological behavior of mice [131]; however, under conditions of cerebral ischemia, the gut microbiota and immune system homeostasis may increase damage to ischemic brain tissue. Crapser et al. found that the intestinal permeability of a mouse model of cerebral infarction increased and was positively correlated with the severity of neurological deficits 72 hours after the onset of cerebral infarction [132]. Telesford et al. analyzed the related literature and found that the gut microbiota affected the systemic immune response; mainly, it was involved in proinflammatory Th1 and Th17 responses against the anti-inflammatory Th2 immune response [133].

The gut microbiota has an important regulatory effect on T cell subsets [121]. When IS occurs, gut microbiota imbalances directly affect Treg and IL-17^+^*γδ*T cells to participate in the process of brain injury [46]; *γδ*T cells secrete a large amount of IL-17, which induces chemotaxis in the surrounding neutrophils and monocytes and aggravates ischemic brain injury [124, 134]. Related studies have shown that, in the late stage of acute brain injury, Treg cells secrete a large amount of the anti-inflammatory cytokine IL-10; this inhibits IL-17^+^*γδ*T cells and produces neuroprotective effects by regulating the peripheral immune system [126,127]. Bodhankar et al. found that increasing the number of CD8^+^ CD122^+^ Treg cells that secreted IL-10 reduced or improved cerebral infarct volume and neurological deficits at 4 days after IS [135]. Singh and colleagues analyzed and found that the number of Treg cells in the peripheral blood of patients with inflammatory bowel disease was significantly less than that of healthy people, while imbalanced gut microbiota inhibited Treg cell differentiation [136].

Winek et al. subjected mice to broad-spectrum antibiotic pretreatment for eight weeks, subsequently blocking the middle cerebral artery [35]. The mortality of the mice increased significantly between days five and seven, which proved for the first time that gut microbiota affected the recovery of acute cerebral infarction. Singh et al. found that IS caused gut microbiota disorder and functional damage, whereas gut microbiota disorder affected the prognosis of IS through inflammation; after gut microbiota balance was restored by the intestinal bacteria, the area of cerebral infarction in mice with middle cerebral artery occlusion was reduced [32].

Houlden et al. found that ischemic brain injury led to changes in the bacterial flora in the cecum of mice; this intestinal change was related to the degree of injury [34]. Sun et al. reported that, by administering *C*. *butyricum* to diabetic mice with brain injury caused by bilateral common carotid artery ischemia reperfusion, neuronal damage was reduced and cognitive function improved [129]. Benakis et al. found that two weeks of amoxicillin and clavulanic acid treatment in mice with acute brain injury reduced the degree of injury by inducing gut microbiota disorder [20].

Lung infection is a common complication of IS. The occurrence of stroke can activate the sympathetic nervous system, increase gastrointestinal permeability, and accelerate the spread and transfer of bacteria to other tissues. When gut microbiota imbalances occur, increased LPS production can affect the tight junctions of the intestinal epithelium through the TLR4/MyD88 signal transduction pathway, upregulating intestinal permeability [137]. Ischemic brain tissue and activated microglia released damage-associated molecular patterns, cytokines, and vagus nerves were activated, which are activated to induce intestinal dyskinesias and intestinal disorders, as well as increase intestinal permeability [138]. Intestinal inflammation and dysfunction caused by cerebral ischemia lead to bacterial infection of organs, while the occurrence of bacterial translocation also increases the area of cerebral infarction [139]. The mechanism of poststroke infection may be sympathetic hyperactivity induced by stroke, anti-inflammatory response mediated by hypothalamic-pituitary-adrenal axis activation, or immunosuppression induced by glucocorticoid hypersecretion [140]. Stanley et al. found that, to promote inflammation after stroke, the intestinal permeability of mice increased, and bacteria moved from the intestinal tract to the lungs, affecting the prognosis [33]. These experimental data imply that, after the occurrence of IS, intestinal microorganisms enter the lungs of mice through the bloodstream and lymphatic systems, causing infection.

Brain ischemia rapidly induces intestinal ischemia and produces excessive nitrate through free radical reactions, resulting in gut dysbiosis with *Enterobacteriaceae* expansion. *Enterobacteriaceae* enrichment exacerbates brain infarction by enhancing systemic inflammation and is an independent risk factor for the primarily poor outcome of patients with stroke. Administering aminoguanidine or superoxide dismutase to diminish nitrate generation or administration of tungstate to inhibit nitrate respiration resulted in suppressed *Enterobacteriaceae* overgrowth, reduced systemic inflammation, and alleviated brain infarction. These effects were gut microbiota-dependent and indicated the translational value of the brain-gut axis in stroke treatment [141] (Tables 1).

### 2.7. Patient Studies Linking Gut Microbiota to IS

In patients with stroke and transient ischemic attack, a decrease in *Bacteroides*, *Prevotella*, and *Enterococcus faecalis*, as well as changes in microbial diversity, was observed [142]. The gut and autonomic nervous systems, certain hormones, and intestinal contents of patients with IS are affected by changes in both the brain-gut axis and gastrointestinal motility [143]. Autonomic nervous system-mediated regulation of mucus secretion plays an important role in the thickness and quality of the intestinal mucus epithelium [144]. When IS occurs, the autonomic nervous system is unbalanced; the gut microbiota “habitat” therefore changes, and gut microbiota imbalance occurs.

Swidsinski et al. found that the resident and pathogenic bacteria in the intestines of patients with stroke changed through dynamic observation [145]. Yamashiro et al. [146] used quantitative polymerase chain reaction technology to observe the gut microbiota of patients with IS, reporting that only *Lactobacillus ruminis* in the gut of patients with IS increased significantly. It was found that the gut microbiota composition in patients with cerebral ischemia differed from that in healthy individuals [107]; *Ruminococcus* increased significantly, while *Eubacterium* and *Bacteroides* decreased significantly.

Studies have found that the gut microbiota and related products are directly involved in platelet activation and thrombosis [111]; others have confirmed that preischemic stress causes gut-derived bacteria to appear in different organs, and bacterial flora shift increases the area of cerebral infarction, reducing the neurological score [139]. Swidsinski et al. analyzed the gut microbiota of patients with cerebral infarction and found that several resident and opportunistic pathogens in the intestinal tract of patients with stroke changed in succession, gradually returning to the control group level after 22 days [145].

The characteristic gut microbiota, especially *Enterobacteriaceae*, might have the ability to predict poststroke cognitive impairment (which is expected to be used as a clinical biomarker) in poststroke patients [147]. Preclinical studies have illustrated the potential role of intestinal bacterial composition in the risk of stroke and poststroke infections. In a prospective case-control study, rectal swabs from 349 patients with ischemic and hemorrhagic stroke were collected within 24 hours of hospital admission. Samples were subjected to 16S rRNA amplicon sequencing, and subsequent alpha and beta diversity analyses revealed higher disruption of intestinal communities during ischemic and hemorrhagic stroke when compared with non-stroke matched control subjects. Aberrations in trimethylamine- and butyrate-producing gut bacteria are associated with stroke and stroke-associated infections [148]. Xia et al. formulated a model stroke dysbiosis index for patients with acute IS based on their gut microbiota dysbiosis patterns. The index was found to be correlated with brain injury and early outcomes, while the model facilitates the potential clinical application of gut microbiota data in stroke and provides quantitative evidence linking the gut microbiota to stroke [149] (Table 2).

### 2.8. Therapeutic Regimens Based on Gut Microbiota

#### 2.8.1. Pre- and Probiotics

New treatment modalities, including both pre- and probiotics, may normalize the gut microbiota composition, change the brain-gut barrier, and decrease the risk of pathological development. Probiotics can hydrolyze proteases to produce peptides with inhibitory activity [150], resulting in a hypotensive effect. A meta-analysis showed that probiotic fermented milk significantly lowered blood pressure in patients with hypertension [151]. After the combined use of antibiotics, the metabolic activity of intestinal microorganisms was inhibited; additionally, the bioavailability of amlodipine increased, which may be an important mechanism for lowering blood pressure [152]. The use of probiotic supplements, high-fiber or acetate diets, and fecal transplantation to restore gut microbiota homeostasis will likely become an innovative strategy for the prevention and treatment of hypertension in the future [153].

According to some studies, enteral nutrition treatment for patients with severe trauma ensures an energy supply, improves the level of gastrointestinal hormones, and promotes the recovery of gastrointestinal and systemic immune functions by regulating the gut microbiota [154]. Recent studies have reported that probiotics can suppress the production of proinflammatory cytokines, such as TNF-*α* and IL-6, following cerebral ischemia. It is well recognized that enhanced proinflammatory cytokines play a critical role in pathophysiological damage following cerebral ischemia, and daily prophylactic ingestion of multispecies probiotics both reduced hippocampal neuronal damage and restored spatial memory performance (in part, by suppressing apoptosis) in a mouse model of cerebral hypoperfusion [155–157].

### 2.9. Fecal Microbiota Transplantation

Fecal microbiota transplantation is a biological therapy that involves transferring the gut microbiota from healthy individuals to patients, with the aim of reconstructing the intestinal microflora of the latter, and is an accepted treatment for recurrent *Clostridioides difficile* infections [158, 159].

Prebiotic treatment exacerbated functional damage and inflammation in a mouse model of stroke, which increased the infarct volume; however, transplantation of healthy microbiota reduced infarct volume [32]. Another study found improved performance in several behavioral tests, decreased mortality and infarct size, and decreased proinflammatory cytokines after fecal microbiota transplantation with young microbiota, compared with those receiving aged microbiota [160].

Poststroke microbiota reconstitution in aged mice using fecal transplant gavage in young mice, which contains higher levels of SCFA-producing bacteria, improves functional and behavioral outcomes. Transplantation of selective SCFA-producing bacterial with the prebiotic inulin reproduces the beneficial effects of young fecal transplant gavage on stroke recovery in aged stroke mice [161].

### 2.10. Chinese Medicine

Traditional Chinese medicine can reduce cerebrovascular damage by remodeling the intestinal microenvironment, weakening bacterial flora translocation, and increasing the use of probiotics. Chen et al. believed that the mechanism of the combined Pueraria and Chuanxiong drug, used to reduce the brain injury of IS through the gut microbiota, was involved in increasing the levels of Claudin-5 and ZO-1; this is reported to regulate internal bacteria such as *Alloprevotella*, Ruminococcaceae, and *Oscillospira*, to remodel the intestinal microecology and weaken the translocation of gut microbiota [162]. Xu et al. found that astragaloside, a traditional Chinese medicine monomer, inhibited the increase of intestinal bacteria such as *Bifidobacterium*, *Megamonas*, *Blautia*, *Holdemanella*, and *Clostridium*, in mice with acute IS [163]. Chen et al. found that resveratrol increased the levels of *Lactobacillus* and *Bifidobacterium* in the intestinal tract of mice, and activity of bile salt hydrolytic enzymes, reduced TMAO, and weakened AS [164]. Zhang et al. reported that berberine in *Coptis chinensis* inhibited the occurrence of obesity and insulin resistance in rats fed a high-fat diet by regulating the structure of gut microbiota and improving the level of SCFAs in the intestine [165]. Wang et al. found that *Polygonatum odoratum* polysaccharides enhanced and improved the species richness and structure of the gut microbiota in rats fed a high-fat diet, improved the level of SCFAs, and, finally, inhibited lipid metabolism and AS [166]. Monomers of Chinese medicine including Paeoniflorin and *Acacia* [167], as well as honeysuckle, *Ganoderma lucidum*, and the Chinese herbal compound Gegen Qinlian Decoction, are all able to treat AS by changing the structure or abundance of gut microbiota [168].

Yu et al. prepared a model of middle cerebral artery ischemia reperfusion in rats with seven days of intragastric rhubarb anthraquinone glycoside administration [169]; the results indicated that the treatment significantly reduced neurological deficit symptoms and infarct area in rats with cerebral ischemia reperfusion. This mechanism may be induced to regulate the gut microbiota of the host, as well as inhibit free radical damage and the inflammatory response caused by brain injury. Seven days of continuous gastric lavage with mixed drugs (Buyang Huanwu Decoction and Enteral Nutritional Emulsion), in cerebral ischemia reperfusion model rats, effectively increased the number of *Lactobacillus* and *Bifidobacterium*, significantly reduced the number of *E*. *coli*, and regulated the proportion of gut microbiota [170]. Studies have shown that Panax notoginseng saponins, with active ingredients similar to ginsenosides, significantly improved cerebral ischemia reperfusion injury [171]; this protective effect enhanced the abundance of *B*. *longum*.

## 3. Conclusion and Prospects

The gut microbiota has become a potential diagnostic and therapeutic target for stroke, Alzheimer's disease, Parkinson's disease, depression, and many other diseases. The gut microbiota affects the occurrence and prognosis of IS by regulating the risk factors that affect IS or related immune responses. IS directly or indirectly affects the gut microbiota by changing the intestinal lumen environment and generating a large number of molecular signals. An increasing amount of research has focused on the mutual regulation between humans and microorganisms, and the relationship between gut microbiota and stroke has become a new area of focus in scientific fields such as microbiology and medical research.

The method still requires improvement based on the existing brain-gut axis concept as due to the complexity of the human gut microbiota and difficulty of research methods, the current understanding regarding the biological mechanism of the gut microbiota and IS remains insufficient. In the future, researchers should aim to fully understand the relationship between gut microbiota and the brain, elucidate the role of gut microbiota in the process of IS, and study how to prevent and treat IS on the basis of microorganisms through the use of probiotics, fecal microbiota transplantation, and Chinese herbal medicine. These studies also require extensive investigation and research including basic and clinical big data regarding IS.

The present research has confirmed that gut microbiota disorder plays an essential role in the pathophysiological process of stroke, including the two-way regulation system between the brain and the intestine, the influence of fecal transplantation and antibiotic intervention on the prognosis of ischemic stroke, etc., which all show the importance and advantages of gut microbiota as the target of treatment of ischemic stroke; however, considering the complexity of the disease, and the specific interaction mechanism between ischemic stroke and gut microbiota has not been clarified, other specific intervention methods such as fecal transplantation still need to be studied, confirmed, and improved by a large number of animal and clinical trials in the further.

## Figures and Tables

**Table 1:** Summary of model studies on gut microbiota in stroke.

Reference	Species	Models	Mechanisms
Benakis et al. 2016 [20]	Mice	Transient focal cerebral ischemia	The revealing of gut-brain axis and the impact of gut microbiota and meningeal IL-17^+^*γδ*T cells on ischemic injury
Singh et al. 2016 [32]	Mice	Permanent distal MCAO model and transient fMCAO model	The gut microbiome was a target of stroke-induced systemic alterations and an effector with substantial impact on stroke outcome
Stanley et al. 2016 [33]	Mice	Ischemic stroke	Stroke promoted the translocation and dissemination of selective strains of bacteria that originated from the host gut microbiota
Houlden et al. 2016 [34]	Mice	Transient focal cerebral ischemia	Brain injury effected the gut microbiota, whereas changes in the gut microbiota after brain injury might affect recovery and treatment of patients
Winek et al. 2016 [35]	Mice	Focal cerebral ischemia	Microbial colonization or specific microbiota were crucial for stroke outcome
Shichita et al. 2009 [124]	Mice	Focal brain ischemia model	T lymphocytes could be a therapeutic target for mitigating the inflammatory events that amplified the initial damage in cerebral ischemia
Liesz et al. 2009 [126]	Mice	Ischemia model	Treg cells were major cerebroprotective modulators of postischemic inflammatory brain damage
Li et al. 2013 [127]	Rodent	Ischemia model	Treg adoptive therapy was a cell-based therapy targeting poststroke inflammatory dysregulation and neurovascular disruption
Sun et al. 2016 [129]	Mice	Diabetes and cerebral I/R	Disruption of the bidirectional interactions between the gut microbiota and the nervous system might be involved in the pathophysiology of acute and chronic gastrointestinal disease states
Crapser et al. 2016 [132]	Mice	Transient focal cerebral ischemia	Compared to young mice, aged mice developed a septic response after stroke
Bodhankar et al. 2015 [135]	Mice	Middle cerebral artery occlusion model	A major neuroprotective role for IL-10^+^ B-cells in treating MCAO
Caso et al. 2009 [139]	Rats	Permanent focal ischemia	Understanding the implication of bacterial translocation during stress-induced stroke worsening was of great potential clinical relevance

MCAO: middle cerebral artery occlusion; fMCAO: filament middle cerebral artery occlusion; I/R: ischemia/reperfusion; IL: interleukin; CNS: central nervous system.

**Table 2:** Summary of patient studies on gut microbiota in stroke.

Reference	Patient group	Participants enrollment	Outcome measures
Zhu et al. 2016 [111]	Plasma TMAO levels in subjects	>4000	The subject's plasma TMAO level independently predicted incident (3 years) risk of thrombosis (heart disease, stroke)
Yin et al. 2015 [142]	Patients with large-artery atherosclerotic ischemic stroke and transient ischemic attack	553	Patients with stroke and transient ischemic attack had more opportunistic pathogens, such as *Enterobacter*, macrophages, *Oscillibacter*, and *Desulfovibrio*, but fewer commensal or beneficial genera, such as *Bacteroides*, *Prevotella*, and *Faecalibacterium*, and had significantly lower TMAO levels
Swidsinski et al. 2012 [145]	Patients hospitalized in two stroke units	110	Typical for stroke was the migration of leukocyte into the mucus within 1–3 days; then, the main fermentative *Roseburia*, *Bacteroides*, and *Faecalibacterium prausnitzii* groups suddenly decontaminated and leukocytes in the stool disappeared; the arrest of bacterial fermentation between within 3–7 days; then *Enterobacteriaceae*, *Bifidobacteriaceae*, and *Clostridium* outnumbered *Bacteroides*, *Roseburia*, and *Faecalibacterium prausnitzii*, and after that declined to initial values
Yamashiro et al. 2017 [146]	Patients with acute ischemic stroke	81	The bacterial counts of *Lactobacillus ruminis* were significantly higher in stroke patients; ischemic stroke was independently associated with increased bacterial counts of *Atopobium cluster* and *Lactobacillus ruminis*, and decreased numbers of *Lactobacillus sakei* subgroup, and was associated with decreased and increased concentrations of acetic acid and valeric acid, respectively; changes in the prevalence of *Lactobacillus ruminis* were positively correlated with serum interleukin-6 levels
Ling et al. 2020 [147]	Patients with ischemic stroke	93	The abundance of *Firmicutes* and its members, including *Clostridia*, *Clostridiales*, *Lachnospiraceae*, and *Lachnospiraceae_other*, was significantly decreased in the age-matched poststroke cognitive impairment group; poststroke cognitive impairment was significantly correlated with the abundance of *Enterobacteriaceae* after adjustments
Haak et al. 2020 [148]	Patients with ischemic and hemorrhagic stroke	400	Disruption of intestinal communities during ischemic and hemorrhagic stroke; an enrichment of bacteria implicated in TMAO production and a loss of butyrate-producing bacteria; twofold lower plasma levels of TMAO; lower abundance of butyrate-producing bacteria within 24 h of hospital admission was an independent predictor of enhanced risk of poststroke infection
Xia et al. 2019 [149]	Patients with acute ischemic stroke	194	Eighteen genera were significantly different between stroke patients and healthy individuals; gut microbiota dysbiosis was significantly correlated with patients' outcome

TMAO: trimethylamine oxide.
